# Book Review

**Published:** 1938

**Authors:** 


### Reviews of Books

**The Doctor's View of War.** By H. Joules, M.D., M.R.C.P. Pp. i., 122. London: George Allen & Unwin Ltd. 1938. Price 3s. 6d.—The authors of this book are recommended by the editor as a well-assorted group, covering a very wide field of medical practice. Unfortunately, the field of war is not included in their medical experience. Their facts are derived from literature, not from experience. However, to do them justice, they have epitomized the official medical history of the War quite well. Milton might have had them in mind when he spoke of "the ferrets and mouse-hunts of an index." Like other sane human beings, they deplore the arbitrament of war, but they have visions of a conference of the Medical Societies of the world pronouncing that for the sake of humanity a system of collective security must be established which might prevent war. As a practical contribution to the promotion of peace this seems rather slender.

**A Synopsis of Physiology.** Third Edition. By A. Rendle Short, M.D., B.Sc., F.R.C.S., and C. L. G. Pratt, M.D., M.Sc. Pp. viii., 325. Illustrated. Bristol: John Wright and Sons Ltd. 1938. Price 10s. 6d.—Physiology is a subject which cannot be curtly summarized with satisfaction; but if this method of treatment is in any degree possible, Rendle Short and Pratt's *Synopsis* certainly makes a brave attempt to present facts and arguments concerning body processes in a concise form, and one must admire the degree of success attained. It is remarkable how much information has been fitted into about three hundred small pages; in this edition the material is reasonably up-to-date in all the important branches of Physiology. The book is probably most useful to students wishing to revise their knowledge of Physiology in a short time; the telegraphic style which is necessary for brevity will probably not worry them, though it would no doubt become irritating to practitioners wishing to keep abreast of the subject, who would fare better with a more expansive text-book. The account of conditioned reflexes is unnecessarily short, and there is no mention of the mechanisms which increase the output of insulin. Amongst the few other criticisms are the consistent misspelling of the word adrenaline, the omission of Benedict's qualitative test in favour of the less sensitive Fehling, an apparent misconception regarding the nature of Mett's tubes and an implication that the *reflex* control of the heart can be studied in the heart-lung preparation (although three pages later it is pointed out that such a heart has lost its normal nervous control) ; and occasionally one comes across bald teleological statements which, after all, do not explain functions. The format and type are excellent and well suited to the purpose of the book.

**Common Happenings in Childhood.** By Sir G. F. STILL, K.C.V.O., M.D., LL.D., F.R.C.P. Pp. x., 180. London: Oxford University Press. 1938. Price 5s.—This book consists of a series of essays or studies of such common events in childhood as crying, laughter, sleep, appetite, tiredness and the like. It is the product of the clinical experience of an outstanding pædiatrician who studied children as well as their diseases. It is not a text-book, but rather a happy blending of scientific observation, philosophic reflection and common-sense advice. No claim is made that any of the subjects are treated either exhaustively or systematically. The book is valuable not so much for what it says, as little of this is new, but for the approach to children that it portrays. It is clearly written and easy to read, so that although primarily intended for doctors and nurses, it can be recommended to anyone who is seriously interested in the welfare of children.

**Arthritis, Fibrositis and Gout.** By C. W. BUCKLEY, M.D., F.R.C.P. Pp. viii., 153. Illustrated. London : H. K. Lewis & Co. Ltd. 1938. Price 7s. 6d.—Dr. Buckley has written a very good book on chronic rheumatic disease and gout. There was every reason why he should have done so, because of his long experience of these diseases in practice and because, as editor of the Reports on Chronic Rheumatic Diseases issued by the British Committee on Rheumatism, he has the opportunity of learning (by discussion and reading) the latest views of other workers in this field. His descriptions and classification are good, whilst his survey of remedial measures is first-rate. This is a most practical book, in which treatment is considered with judgment and described with concise but adequate detail. It will be found a very useful guide in practice.

**Reports on Chronic Rheumatic Diseases. No. 4.** By C. W. BUCKLEY, M.D., F.R.C.P. Pp. xii., 155. Illustrated. London: H. K. Lewis & Co. Ltd. 1938. Price 10s. 6d.—This volume is the last annual report of the British Committee on Chronic Rheumatic Diseases. The work has now been taken over by the Scientific Advisory Committee of the Empire Rheumatic Council, and in future reports will appear more frequently. This fourth report contains many and varied articles, mainly on treatment. Professor Stanley Davidson opens with a discussion on the social aspects, maintaining that the voluntary hospitals are unable to cope with the problem. Dr. Philip Hench of the Mayo Clinic writes on how they tackle arthritis and rheumatism in the U.S.A. Dr. Mathieu-Pierre Weil, of Paris, contributes an interesting article on radio-active substances and the treatment of rheumatism. There are critical reviews on vaccine treatment and on gold therapy. Professor Mitchell, of Edinburgh, illustrates comparative clinical and pathological features in "Rheumatic Disease" in the horse. One of the most comprehensive chapters is that by Dr. Kersley of Bath on "The Causation and Treatment of Sciatic Pain." The final chapter is a useful but all too brief survey of the physiological action of some physical methods by Sir Leonard Hill and Dr. H. J. Taylor.

**Heart Disease and Pregnancy.** By C. BRAMWELL, M.D., F.R.C.P., and E. A. LONGSON, M.B., Ch.B., D.P.H. Pp. xii., 194. Illustrated. London: Oxford University Press. 1938. Price 8s. 6d.—This book is the record of 350 cases of organic heart disease in pregnancy studied by the authors, together with the conclusions reached as a result of this study. All but twenty-five of the cases were observed during the ante-natal period, throughout labour and the puerperium, and many of them have been followed up to date. Not the least interesting result of this study is the conclusion that "although auricular fibrillation does undoubtedly add to the risk of pregnancy, it does not constitute a bar to pregnancy, provided that the patient can be kept under observation and receive adequate treatment." This book serves a most valuable function in that it gives sound information on a most important and difficult subject. Providing as it does a complete guide to the investigation, management and prognosis, both immediate and late, of heart disease in pregnancy, it should find a place on the bookshelves of all practitioners interested in midwifery.

**A Guide to Anatomy.** Fourth Edition. By E. D. EWART. Pp. xii., 342. Illustrated. London: H. K. Lewis & Co. Ltd. 1938. Price 12s. 6d.—This book, by a non-medical author, is written especially for the use of students of massage, and that it has fulfilled its purpose in the past seems to be indicated by the fact that the edition under notice is the fourth. It contains an enormous amount of information on the subject with which it deals, necessarily condensed but sufficiently full for the purpose. In fact, it contains so much that one cannot help sympathizing with the student who has to learn so much detail of a difficult subject. The facts are presented in a simple way, and the accuracy of the statements made is such that not a single error can be found after a very close scrutiny. The illustrations are well-chosen and adequate for the purpose, and the index is complete. Altogether it may be said that this book should provide the student of massage with all the anatomical knowledge she requires, presented in a way which could not be bettered.

**Medicine for Nurses.** By C. B. PERRY, M.D., F.R.C.P. Pp. xi., 211. Edinburgh: E. and S. Livingstone. 1938. Price 5s.—This book is of convenient size, clearly and well printed. The general arrangement of the subject-matter is good. The preface states that the book is intended to aid study "for the Final Examination of the General Nursing Council without describing diseases in detail." Facts, therefore, often seem to be dealt with in a vague and inadequate manner, *e.g.*, page 164, Anterior Poliomyelitis. The pathological lesion is not described, nor is the type of paralysis mentioned. It would seem that much space has been given to subjects already dealt with in text books for the Preliminary Examination, *e.g.* Parasites. It is a pity that no illustrations likely to be helpful to senior nurses are included.

